# Patterns of sexually transmitted infections in patients presenting in special treatment clinic in Ibadan south western Nigeria

**DOI:** 10.11604/pamj.2015.21.222.6056

**Published:** 2015-07-30

**Authors:** Victor Ugochukwu Nwadike, Olawale Olusanya, Gloria Chinenye Anaedobe, Iche Kalu, Kingsley Chiedozie Ojide

**Affiliations:** 1 Department of Pathology, Federal Medical Centre (FMC) Abeokuta, Ogun State, Nigeria; 2 Medical Microbiology, Pathcare Nigeria, Lagos, Lagos State, Nigeria; 3 Special Treatment Clinic, Department of Medical Microbiology, University College Hospital Ibadan, Oyo State, Nigeria; 4 Department of Medical Microbiology, Federal Medical Centre, Umuahia, Abia State; 5 Department of Medical Microbiology, Federal Teaching Hospital Abakaliki, Ebonyi State

**Keywords:** STI, STC, IBADAN

## Abstract

**Introduction:**

Sexually transmitted infections (STIs) are infections that are often transferred from one person to another during sexual activity. In developing countries, an increase in the incidence of STIs is attributed to increasing urbanization, modernization, travel, education and exposure to Western media which has led to increased sexual activity, especially among young people.

**Methods:**

This is a retrospective study carried out in the University College Hospital (UCH) Ibadan, Nigeria. The records of a total of 506 patients who attended the clinic between Jan 2010-Dec 2011 were retrieved. The records of the patients’ complaints were taken. Detailed demographic data and history of genital symptoms was taken.

**Results:**

The records of 506 patients were used 43.7% (221) were males and 56.3% (285) were females. The patient's age ranged from one to eighty, the 1-10 age groups and the 71-80 ages were the least represented age group. Age, sex, level of education, presenting complaints, presence of yeast cells, VDRL positivity were variables that were looked at. Of these only sex and occupation were risk factors for transmission of STI.

**Conclusion:**

Good clinical care for patients with STIs should extend beyond therapy and include help to avoid future infections. Control activities should focus on the primary prevention of infection through safer sexual practices. Strategies for improving secondary prevention (health care-seeking behavior and case management) should include identification of people at risk and targeting them for intervention.

## Introduction

Sexually transmitted Infections (STIs) are infections that can be transferred from one person to another during sexual activity. They also involve the transmission of pathogens from one person to another during sexual contact. STIs could be either ulcerative or non-ulcerative. In developing countries, STIs exhibit a higher prevalence rates than developed countries. [1] The World Health Organization (WHO) postulates that approximately 340 million new cases of the four main curable sexually transmitted infections (STIs) gonorrhea, Chlamydia infection, syphilis and trichomoniasis occur every year, 75-85 per cent of them in developing countries [2]. Moreover, the interest in STIs and their management have increased tremendously because Of their proven role in facilitation of HIV infection [2], this in turn increases susceptibility to other STIs. An upsurge in the incidence of STIs in developing countries has been linked to increasing urbanization, modernization, travel, education and exposure to Western media. This has increased sexual activity, especially among young people [3]. STIs are often asymptomatic, particularly in women.[3] Identified risk factors for STIs include multiple sexual partners (premarital and extramarital sex), inconsistent use of condoms and sexual preferences.[3] Previous STI, exposure to a symptomatic partner and suppressed immunity are also recognised predisposing factors.[4] STIs and their complications cause substantial morbidity and mortality which is independent of their role in the transmission of human immunodeficiency virus (HIV).[4] The profile of the various diseases is variable depending upon the socio-economic, cultural, geographic and environmental factors prevalent in different parts of the country [5]. However in a study done in india it was shown that because of lack of adequate laboratory infrastructure in the country, data available from all the regions are usually based on syndromic diagnosis[6]. It is important to have baseline information on the epidemiology of STIs, the proportion of symptomatic and asymptomatic infections and other associated risk behaviors’ for the designing, implementation and monitoring of targeted interventions programs [7]. Clinical care for patients with STIs should extend beyond treatment. Control measures to prevent future infections should be a cornerstone in the management of STI. Control activities should focus on the primary prevention of infection through abstinence, sticking to monogamous relationships, safer sexual practices, including condom use.[3] Other Strategies should include identification of people at high risk of acquiring and transmitting infections and targeting them for intervention.[4] The aim of the present study was to document the pattern of common STIs and to evaluate the frequency of occurrence of STIs among STC attendees in Ibadan, South western Nigeria.

## Methods

This is a retrospective study carried out in the University College Hospital (UCH) Ibadan, Nigeria. The University College Hospital is an 850 bedded tertiary health institution with a WHO reference center for the treatment of sexually transmitted infections. The study population consists of patients who attended the STC clinic. The records of a total of 506 patients who attended the clinic between Jan 2010-Dec 2011 were retrieved and data used for the study. In these patients genital examination was performed, urethral, vaginal and endocervical swabs were taken as the case may be for microscopy, culture and sensitivity, blood for VDRL serology was also taken. The specimens were processed in the on-site hospital laboratory using standard methods. The specimens were inoculated on selective and non selective media. The specimen was inoculated on MacConkey agar/ blood agar and incubated at 35-37oC for 18-24hrs in CO2 enriched environment. Statistical analysis: all data were analyzed using the Statistical Package for the Social sciences (SPSS) version 15.0. Data were presented using frequency tables, charts, as appropriate and cross tabulation to study relationships and association between variables. Statistical significance was set at 5%.

## Results

A total of 506 patients were recruited into the study, 43.7% (221) were males and 56.3% (285) were females. Male to female ratio was 1:0.8. The patient's age ranged from one to eighty, the 1-10 age groups and the 71-80 ages were the least represented age group (Table 1). All the patients had some level of education, those with primary level education being 8.5% (43), secondary education 36% (182) and tertiary education 55.5% (281).Table 1 Occupation of the clients in the clinic ranged from self-employed people to commercial sex workers, 31.2% (158) were self-employed, and 0.2% (1) was commercial sex patients. Table 1 Majority of the patients presented with vaginal discharge 26.9% (136), other modes of presentation were urethral discharge, genital warts, genital ulcer, genital itching and one case of rape (Table 2). The following diagnosis was made in the following order candidiasis 18.2% (92), genital warts 14.8% (72), genital ulcer disease 12.1% (61), pelvic inflammatory disease 6.5% (33), non-gonococcal urethritis 4.5%(23), gonorrhea 3.4%(17)(Table 3). Three patients (0.6%) who presented in the clinic had laboratory identified gonorrhea Table 3. 10.9% (55) had yeast cells in the microscopy and cultures 29.6% (150) had pus cells on microscopy (Table 1).

**Table 1:** Frequency table of demographic variables

Variables	Sub Variables	Frequency	Percentage (%)
Sex	Male	221	43.7
	Female	285	56.3
Age	1-10	3	0.6
	11-20	27	5.3
	21-30	253	50.0
	31-40	142	28.1
	41-50	56	11.1
	51-60	19	3.8
	61-70	5	1.0
	71-80	1	0.2
Level of Education	Primary	43	8.5
	Secondary	182	36.0
	Tertiary	281	55.5
Occupation	Student	147	29.1
	Corper	18	3.6
	Self employed	158	31.2
	Unemployed	55	10.9
	CSW	1	2
	Civil Servant	116	22.9
	Uniform Men	6	1.2
	Driver	5	1.0
Total		506	100

**Table 2:** Frequency table of Clinical variables and findings

Variables	Sub variables	Frequency	Percentage (%)
Presenting Complaints	Vaginal Discharge	136	26.9
	Urethral Discharge	56	11.1
	Genital Ulcer	44	8.7
	Genital Warts	36	7.1
	Genital Itching	58	11.5
	Dysuria	22	4.3
	Rape	3	0.6
	Others	151	29.8
Presence of Gonococci	Positive	3	0.6
	Negative	503	99.4
Presence of Pus cells	Positive	150	29.6
	Negative	356	70.4
Presence of yeast Cells	Positive	55	10.9
	Negative	451	89.1

The above is a frequency table showing the frequencies of selected variables

**Table 3:** Relationship between level of education and diagnosis

LOE	Genital ulcer disease	Genital warts	Gonorrhea	Non Gonococcal Urethritis	Bacterial vaginosis	Candidiasis	Pelvic Inflammatory disease	Tinea
Primary	5(8.2%)	4(5.3%)	2(11.8%)	2(11.1%)	2(11.1%)	9(9.8%)	4(12.1%)	2(16.7%)
Secondary	19(31.1%)	25(33.3%)	8(47.1%)	8(26.1%)	7(38.9%)	35(38%)	12(36.4%)	4(33.3%)
Tertiary	37(60.7%)	46(61.3%)	7(41.2%)	15(65.2%)	9(50%)	48(52.2%)	17(51.5%)	6(50%)

X = 0.965. The table shows the relationship between level of education and the diagnosis that was made

Majority of patients who presented with genital warts were males 72% (44) compared to 28% (17) who were females, 39%(29) were managed for gonorrhea compared to 61%(46) who were females. Sex was an important factor in STI acquisition. X = 0.000 Rate of STI was higher in the group who had tertiary education {Genital ulcer disease 60.7%(37), Genital warts 61.3%(46), Gonorrhea 41.2%(7), Non Gonococcal Urethritis 65.2%(15)} compared to Genital Ulcer Disease 31.1%(19), Genital warts 33.3%(25), Gonorrhea 47.1%(8) and 8.2%(5) GUD, 5.3%(4) Genital warts, 11.8%(2) Gonorrhea in people with secondary and primary education. Level of education did not significantly affect transmission of STI (Table 3). When occupation was cross tabulated against diagnosis most of the patients who presented in the clinic were self employed {(GUD-34.4% (21), genital wart-24% (18), Gonorrhea-(29.4%(5)}, the presentation in this group was higher than in any other group. Occupation of the patient affects the transmission of STI (X = 0.017) (Table 4). Only 2(0.4%) of the 506 patients were VDRL positive, 50% (1) of the patients were male and 50% of them females. The two people who tested VDRL positive were in the self employed and unemployed groups. Fifty percent (1) had secondary education and another 50% (1), level of education did not affect the transmission of STI. Two (66.7%) of the three patients who had laboratory diagnosis of gonorrhea were male compared to just a female who had gonorrhea. Two (66.7%) of the patients had secondary education, one (33.3%) had university education and none had primary education and fifty five (10.9%) of the patients on microscopy of their specimens had yeast cells.

**Table 4:** Relationship between occupation and diagnosis

Occupation	Genital Ulcer Disease	Genital Warts	Gonorrhea	Non Gonococcal urethritis	Bacterial vaginosis	Candidiasis	Pelvic Inflammatory disease	Tinea
Student	11(18%)	27(36%)	7(41.2%)	8(34.8%)	6(33.3%)	29(31.5%)	10(30%)	2(16.7%)
Corpers	3(18%)	5(6.7%)	(0%)	3(13%)	0(0%)	2(2.2%)	0(0%)	0(0%)
Self employed	21(34.4%)	18(24%)	5(29.4%)	7(30.4%)	5(27.8%)	32(34.8%)	11(33.3%)	6(50%)
Commercial sex workers	0(0%)	0(0%)	0(0%)	0(0%)	1(5.6%)	0(0%)	0(0%)	0(0%)
Unemployed	8(13.1%)	13(17.3%)	2(11.8%)	0(0%)	2(11.1%)	7(11.1%)	3(9.1%)	2(8.3%)
Civil servant	15(24.6%)	12(16%)	2(11.8%)	4(17.4%)	4(22.2%)	22(23.9%)	9(27.3%)	1(8.3%)
Drivers	1(1.6%)	0(0%)	0(0%)	1(4.3%)	0(0%)	0(0%)	0(0%)	1(8.3%)

X = 0.017. The above is a frequency table showing the relationship between occupation and diagnosis

## Discussion

Available data from a previous study show that sexually transmitted infections constitute great medical, social and economic problems in Nigeria (8). In fact in 1963, WHO found Lagos to have the highest gonorrhea rate in the world. Recent surveys report gonorrhea prevalence to be as high as 28.1% [8]. In the present study, most of the STI cases occurred in the 21-30 yr age group, this is similar to that reported in other studies in Ibadan Nigeria by Okonko et al [9]. This age group is the sexually active group and at a high risk of being behaviorally more vulnerable to STI acquisition, as they generally have higher number of sexual partners and more concurrent partnerships and change partners more often than older age groups [10]. Being the economically productive group, there is a great loss of manpower at work due to STI morbidity [11]. This is also the predominant age group observed to be having STI cases in other Indian studies [12]. Prevalence of STI is as follows in this study Gonorrhea 3(0.6%), Candida 55(10.9%) and syphilis 2(0.4%). This is lower than what was reported by Ikimalo et al in Nigeria [13], where the rates were Gonorrhea 2.1%, Syphilis 1.0%. This may be due to the fact that this study was conducted in a single location against the other which was a multi center study. In the present study Candida albicans was found in 55(10.9%) of the respondents this differs from studies done by okonko et al in Nigeria where candidiasis was the commonest STI in the women who present in the STC clinic. A marked decline in bacterial STIs resulting in an apparent increase in fungal and viral STI have been reported from different regions (Okonko et al) [9]. More women 221(43.7%) presented in the STI clinic than males 285 (56.3%), this differs from reports in India where the reverse is the case [14].

Genital ulcer disease was responsible for 44(7.1%) of STI in the STC clinic for the study period, this is higher than results reported by Fatiregun et al in Ibadan and this is important because of the association between HIV and genital ulcer disease as it has been discovered that Genital ulcer disease increases the predilection for HIV [15]. Majority of the patients who presented in the STC clinic were self employed closely followed by students, this is different from what was reported by fatiregun et al where the highest presentations were in students. This increase in population of self employed seeking care may be because of the perceived economic power of the patients they may indulge in risky sexual behavior [15]. In this study the largest users of the STC were people with tertiary education this differs from reports from studies in ile Ife where most of the participants had primary education. The high rate of tertiary educated participants seeking care in this study may be due to the increased awareness about STI in the society and also its link as a cause of infertility. In conclusion, this study has shown a relatively high prevalence of STIs. The control measures for STI should target risky sexual behaviours in the study community. Targeting high-risk individuals such as commercial sex workers, their clients and adolescents is an important STI control strategy as seen from this study when only one commercial sex worker presented in the clinic and was treated for bacterial vaginosis. In view of the fact that STIs and sexual behaviours have been found to be independently associated with HIV transmission, there is a great need for effective control of STIs to reduce HIV transmission.

## Conclusion

Focused health education and proper counseling to change risky sexual behaviors constitute important STI control measures in addition to early recognition and prompt and adequate treatment. The clinical microbiologist has an important role to play in all these STI control strategies.
